# Sinonasal desmoplastic small round cell tumor: a case report and review of the literature

**DOI:** 10.1186/s12885-019-6076-4

**Published:** 2019-08-31

**Authors:** Yanli Tao, Lina Shi, Li Ge, Tiejun Yuan, Li Shi

**Affiliations:** 1 Department of Otolaryngology, The Second Hospital of Shandong University, 247#, Beiyuan Street, Jinan, 250033 China; 2 Department of Otolaryngology, Weifang People’s Hospital, No. 151, Guangwen Street, Kuiwen District, Weifang, 261000 China; 3 Shandong Provincial Key Laboratory of Oral Tissue Regeneration, Department of Bone Metabolism, School of Stomatology, Shandong University, Wenhua West Road 44-1, Jinan, 250012 China; 4 Department of Pathology, Weifang People’s Hospital, No. 151, Guangwen Street, Kuiwen District, Weifang, 261000 China

**Keywords:** Desmoplastic small round cell tumor, Surgical resection, Multimodal management

## Abstract

**Background:**

Desmoplastic small round cell tumor (DSRCT) is a rare malignancy with poor prognosis that generally involves the peritoneum. Its diagnosis can be achieved only by immunohistochemistry and cytogenetic studies.

**Case presentation:**

In the current report, a 55-year-old female was admitted in our hospital for evaluation of right eye epiphora and right nasal intermittent bleeding. Imaging examination revealed a large soft tissue mass in the right nasal cavity and ethmoid sinus. After an explorative surgery, the pathological findings confirmed the presentation of sinonasal DSRCT. Immunohistochemistry and cytogenetic studies confirmed the diagnosis of DSRCT in this patient. Surgical resection, chemotherapy, and radiotherapy was performed, and she died 2 months after operation.

**Conclusion:**

This reported case draws attention to the importance of novel treatments and including DSRCT in the differential diagnosis of sinonasal tumors.

## Background

Desmoplastic small round cell tumor (DSRCT) is a rare and aggressive mesenchymal malignancy. Only 850 such patients were reported in the medical literature [1]. First described in 1989 [2, 3], DSRCT’s name derives from its distinctive histological findings, which include clusters of undifferentiated, small round blue cells surrounded by abundant desmoplasia.

Patients with DSRCT are usually between 5 and 30 years of age. Males and older adolescents are more often affected [4, 5]. The most commonly affected region is the pelvis, other sites mainly include the omentum, the spatium retroperitoneale and the mesentery [6–8]. Tumors located in the abdomen or pelvis generally require a period of growth before they can cause symptoms of the body. When the symptoms of the body appear, it is usually atypical, mainly characterized by abdominal pain, weight loss, abdominal fullness, etc. Therefore, DSRCT is often diagnosed when the tumor has metastasized or is in the advanced stage of the disease. According to the SEER database, only about one-fifth of patients are diagnosed with only localized disease [4].

In spite of great advances in medical technology, the treatment of DSRCT remains a challenge for doctors. There are currently no standardized treatment approaches. Surgical resection combined with chemotherapy and radiotherapy are the main treatment methods at present [5]. Surgery is currently the best treatment option. The 3-year survival rate for complete tumor resection cases was reported to be 58%, compared to 0% in unresectable cases [9]. However, surgery does not produce any benefit for patients with extraperitoneal metastases [10]. DSRCT still has a poor prognosis despite these multimodal treatments, with a 3-year survival rate of less than 30% and a 5-year survival rate of only 18% [11, 12]. Therefore, novel therapy is required.

We present a recent case of sinonasal DSRCT and review the literature. The purpose of this study is to describe the microscopic patterns and cytological criteria as well as to describe our experience in the diagnosis and treatment of DSRCT.

## Case presentation

**Fig. 1:**
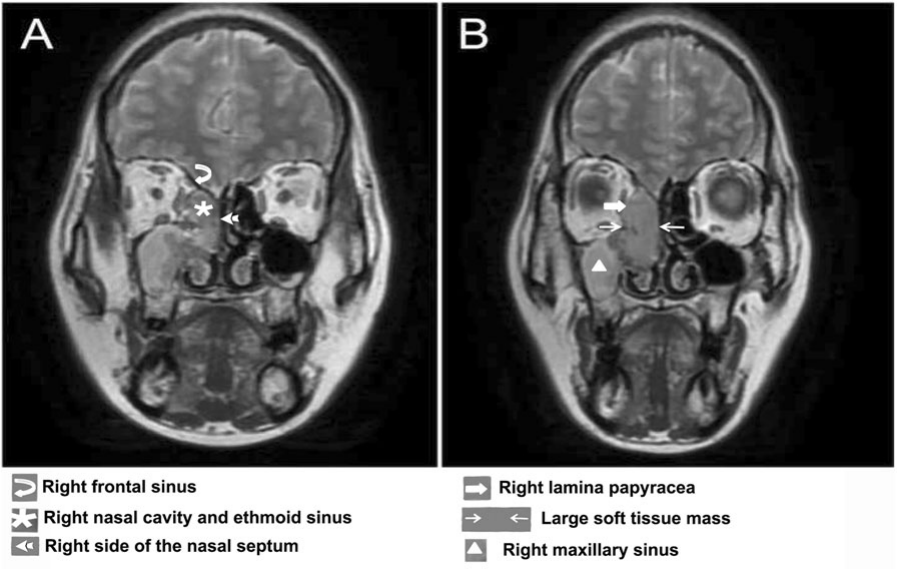
Imaging changes of the patient before surgery. Large soft tissue mass located in the right nasal cavity and ethmoid sinus, invading the right lamina papyracea, right frontal sinus, right side of the nasal septum. The medial wall of right maxillary sinus, the ethmoid cornua, and part of the ethmoid sinus were observed with erosional bone resorption and destruction

A 55-year-old female was admitted in our hospital for right eye epiphora and right nasal intermittent bleeding on August 2018. Nasal endoscopy revealed a right nasal mass located in the middle nasal meatus. A magnetic resonance imaging (MRI) and computed tomography (CT) scans showed a large soft tissue masse in the right nasal cavity and ethmoid sinus, which invaded the right lamina papyracea, the right frontal sinus and the right side of nasal septum. The medial wall of the right superior collar sinus, middle turbinate and part of ethmoid sinus septum were accompanied by erosive bone resorption (Fig. 1a-b). Swollen lymph nodes can be seen in the right neck. The patient underwent endoscopic biopsy of the right ethmoid sinus and pathological examination. Fragments of soft to firm gray and tan tissue were submitted for pathologic examination. The sinus tumors in the right nasal cavity were resected under nasal endoscope.

Diagnosis required confirmation of histopathological features, polyphenotypic immunohistochemical reactivity, and molecular/cytogenetic findings [13], therefore the following experiments were performed.

### Histopathological manifestations

**Fig. 2:**
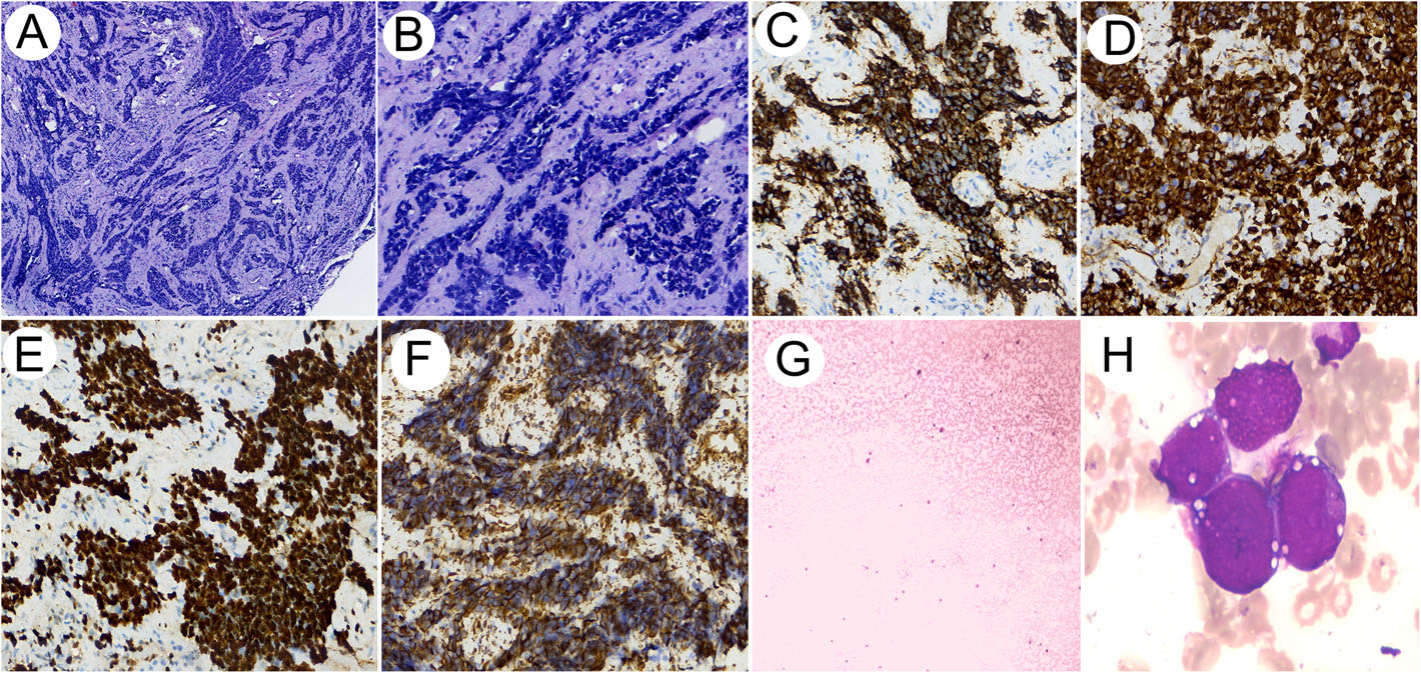
**a**-**b** HE morphology of the patient. Tumor cells were irregular sheet-like and nest-like distribution, surrounded by proliferative fibrous stroma. The tumor cells in the nest are small round or oval, with few cytoplasm, unclear cell boundaries, round or oval hyperchromatic nuclei, unclear nucleoli, and mitotic figures were easy to observe (10× and 40×). **c**-**d** Desmoplastic small round cell tumor shows immunoreactivity for CD56 (40×) and WT-1 (40×). **e** Desmoplastic small round cell tumor shows positive Ki-67 with an index of 95% (40×). **f** Vim stain revealed the characteristic dot-like perinuclear pattern (40×). **g** Blood smear showed no obvious increase or decrease of white blood cells, normal neutrophils, and middle and late red blood cells account for 6/100 nucleated cells, and platelets were scattered. **h** Bone marrow smear image. The cancer cells were scattered or clustered. Their cell bodies were large with unclear boundaries and a large amount of cytoplasm, stained with purple-blue or purple-red, partially foamy, with large nuclei and chromatin accumulation. Naked nuclear tumor cells were frequently observed

Under the light microscope, the lesions were composed of irregular lamellae and nested tumor cells and the surrounding fibrous interstitial cells. The tumor cells in the nest are small round or oval, with few cytoplasm, unclear cell boundaries, round or oval hyperchromatic nuclei, unclear nucleoli, and mitotic figures were easy to observe (Fig. 2a-b). The stroma is a proliferative dense fibrous connective tissue composed of fibroblasts and myofibroblasts with mucoid degeneration. In this case, the tumors invaded bone.

### Immunohistochemical staining

Immunohistochemistry gave the following phenotypic markers: CD56 (+) (Fig. 2c), Vimentin (+), WT-1 (+) (Fig. 2d), S-100(−), Desmin (−), CD99 (−), with a Ki-67 index of 95% (Fig. 2e). Vim showed characteristic dot-like perinuclear staining pattern (Fig. 2f), and did not express EMA, Desmine, S-100, MyoDl, CD20, CD3, CD7 and P63.

### Bone marrow puncture results

Blood smear: There is no obvious increase or decrease of white blood cells, neutrophils are almost normal, and middle and late red blood cells account for 6/100 nucleated cells, and platelets are scattered. (Fig. 2g).

Bone marrow smear: Bone marrow proliferation was active, G = 49.0%, E = 21.0%, G/ E = 2.33:1. The main stage of granulocytes was below the middle and young granulocytes, with no obvious morphological abnormalities. All stages of the erythroid system were noticed, there was no obvious abnormality in morphology, and the size of mature red blood cells was uneven. The cancer cells were scattered or clustered. Their cell bodies were large, the boundaries were unclear, round or irregular, with a large amount of cytoplasm, stained with purple-blue or purple-red, partially foamy, with large nuclei and chromatin accumulation. Naked nuclear tumor cells were frequently observed. Plasmacytes and histiocytes were easy to observe. No megakaryocytes and thrombocytopenia were found in the whole smear. (Fig. 2h).

### Treatment and outcome

During the operation, a small part of the tumors were removed by forceps and sent for rapid frozen pathological diagnosis. The results showed that the tumors were small cell malignant tumors. After the uncinate process was removed, the maxillary sinus was opened and the purulent secretions in the maxillary sinus were sucked out. The maxillary sinus orifice and the medial wall of the maxillary sinus were removed and sent to pathology. The pathological results showed that the orbital cardboard was partially absorbed. Then the orbital cardboard and the orbital fascia were fully separated and removed. After resection of the right frontal sinus mass, the right middle turbinate was removed. It was found that the nasal cavity mass was grey-white fish-like and invaded the right frontal sinus, the right orbital cardboard and the medial wall of the right maxillary sinus. The orbital fascia was intact. Postoperative pathology showed that the tumors were found in the right orbital wall, the right maxillary sinus wall, the posterior wall, the posterior inferior wall and the right middle turbinate root. Chemotherapy and radiotherapy was performed, but the patient was found to have bone marrow metastasis and presented with persistent nasal bleeding and she died 2 months after operation.

## Discussion and conclusion

DSRCT usually occurs between 5 and 50 years with an average age of 22 years. In general, nearly 85–90% of patients are male, but in patients younger than 20 years old at the time of diagnosis, the proportion of females is slightly higher [9]. The clinical manifestations of DSRCT are not typical, and patients often experience abdominal or pelvic discomfort, typically including abdominal pain and/or bloating, ascites, constipation, and urinary tract disease [14–16].

DSRCT, which occurs in the nasal cavity and sinuses, is extremely rare, and only two cases reported in the literature were retrieved on PubMed, their clinical features, treatment and outcomes were summarized in Table 1. Its clinical manifestations are complex and varied. Generally, local symptoms occur according to different parts of the body, without its own unique clinical manifestations. If it occurs in the abdominal cavity or pelvic cavity, clinical manifestations are usually abdominal pain, abdominal distension or abdominal mass, which can be accompanied by cachexia such as fever, anemia, emaciation, and prone to substantial organs and lymph node metastasis; while in the nasal cavity and paranasal sinuses, the primary symptoms are mainly sinusitis, nosebleeds and nasal congestion, local infiltration and cervical lymph node metastasis may occur, and small round cell malignant swelling is common in this area. The clinical manifestations of the tumors were not significantly different, and it was difficult to make a definite diagnosis in clinic.

**Table 1:** Case reports of sinonasal desmoplastic small round cell tumors reported in the literature

Author	Age (y), Gender	Main symptom	Tumor location	Treatment	Patient outcome at time of report
Present study	55, F	Right eye epiphora, right nasal intermittent bleeding	Right ethmoidal sinus, frontal sinus and lamina papyracea	Tumor resection	Survival of 12 mo
Fink MD. et al. [17]	21, F	Chronic sinusitis	Frontal, ethmoidal and sphenoid sinus	Tumor resection; Radiotherapy; Chemotherapy	Survival of more than 26 mo
LOPEZ F. et al. [18]	61, M	Stuffy and bleeding of right-side nose occasionally	Right ethmoidal sinus and anterior cranial fossa	Tumor resection; Radiotherapy	Survival of more than 29 mo

*F* Female, *M* Male

Despite multimodal therapy, patients with DSRCT overall have very poor survival rates of 15–30% at 5 years [4, 5]. The case in this report died 2 months after surgery because of bone marrow metastasis of the tumor, which may be one of the reasons for the poor prognosis in the current case compared with the cases reported in the literature.

The majority of desmoplastic small round cell tumors can be reliably diagnosed based on the characteristic morphology and immunohistochemical profile. Most literatures reported that DSRCT cells expressed epithelial, mesenchymal and neuroendocrine markers [19]. However, some reports showed that the immunophenotype of some DSRCTs were atypical, only expressed Vim, CD56 and other markers, but did not express epithelial and neurogenic or myogenic markers [20]. Des and NSE were not expressed in this case, but only Vim, CD56 and WT-1 markers, which made it difficult to diagnose and differential diagnose. Therefore, familiarity with the characteristic immunophenotypes and molecular pathological changes of DSRCT will be helpful in differential diagnosis.

Due to its histological similarities with other malignant ‘small’ round cell tumors, DSRCT has been confused histologically with other lesions, including primary olfactory neuroblastoma [21], small-cell anaplastic carcinoma and Ewing sarcoma /primitive neurotodermal tumor (PENT). Olfactory neuroblastoma usually occurs in olfactory cleft. Comparison between DSRCT and other small round cell tumors were shown in Table 2. Neurogenic markers such as NSE and synaptic vesicle protein were strongly positive in tumor cells, while low molecular weight keratin was weakly expressed in only a few cases. Myogenic and epithelial antigens were not expressed and S100 was expressed in sertoli cells around the tumor nest. These clinical and pathological features as well as molecular pathological examination are helpful in differentiating DSRCT. Secondly, it is necessary to identify small-cell anaplastic carcinoma. The tumor cells express epithelial markers, partially express neuroendocrine markers, but lack obvious multi-directional differentiation, do not express myogenic markers, and have few interstitial. The dot-like expression of Vim in DSRCT tumor cells is of great value in differential diagnosis [22]. The third is extraosseous Ewing sarcoma /primitive neurotodermal tumor (PENT), which is predominantly located in the lower extremities, spine, retroperitoneum and pleura. It can also occur in the nasal cavity and paranasal sinuses. Its onset age, histological morphology, immunophenotype and molecular pathological changes overlap with DSRCT to a certain extent. When Ewing sarcoma /PENT tumors contain a large amount of fibrous connective tissue, it is very easy to be misdiagnosed as DSRCT [23]. However, the former generally does not express epithelial or myogenic markers, CD99 is strongly positive, and the molecular pathological changes are EWS-FLI1 gene fusion. These features can help differential diagnosis. In this case, WT-1 (+), CD99 (−) can exclude extraosseous Ewing sarcoma and neuroblastoma.

**Table 2:** Comparison between DSRCT and other small round cell tumors

Item	DSRCT	Extraosseous Ewing’s sarcoma/primitive neuroectodermal tumors (PNET)	Olfactory neuroblastoma	Small cell undifferentiated carcinoma
Morphological characteristics	Nests of small round cells vary in size and shape, and there are a large number of fibrous connective tissue stroma between the nests of tumor cells. Tumor cells are closely arranged, thin and sparse, with unclear cell boundaries, round or oval nuclei, hyperchromatic nuclei, unclear nucleoli, and mitotic figures are easy to see	Round cells are compactly patchy/lobular, and fibrovascular septa are observed between lobules with varying width of fibrous connective tissue. The cytoplasm of the tumor cells is scarce and unclear, but some of the cytoplasmic margins could be bright or vacuolar. The nuclei are round/oval, dark-stained/uniform pepper-salt-like, and the mitotic figures vary.	Round cells are nested/lobulated, and interlobular spaces are vascular-rich fibrous connective tissue. Tumor cells differentiate in different degrees. The well-differentiated nuclei of tumor cells have no obvious atypia, fine chromatin, no obvious nucleoli, few mitotic images and more nerve fiber networks in the interstitium. The poorly differentiated tumors have obvious nuclear atypia, easily seen mitotic images, few/absent interstitial fibrous networks and a large number of necrosis.	Small round cells without specific morphology
Immunohistochemistry	Multidirectional differentiation and positive expression: epithelial marker, neuroendocrine marker, WT-1, Desmin (paranuclear point positive), Vimentin (paranuclear point positive); negative: CD99	Positive expression: CD99, Vimentin, CyclinD1; Different degrees of expression: neuroendocrine markers; No expression: epithelial markers, WT-1, Desmin, S-100, NF	Positive expression: neuroendocrine markers (such as NSE, Syn), NF, GFAP, S-100 (sertoli cells around the cancer nest +), epithelial markers (a few weak expression of low molecular keratin); No expression: Desmin, EMA, CD99	Positive expression: epithelial markers; Partial expression of neuroendocrine markers; lack of multidirectional differentiation; Negative expression: Desmin et al.
Molecular pathology	EWS-WT1 gene fusion	EWS-FLI-1 gene fusion	No specific molecular changes	No specific molecular changes
Clinical features	> 95% occurred in pelvic and abdominal cavity, < 5% in paranasal sinuses, pleura, testis, intracranial, liver, lung, mediastinum, ovary, pancreas, etc	Usually occurs in lower limbs, spine, retroperitoneum, pleura, etc. and can also occur in nasal cavity and paranasal sinuses	Prevalent in upper turbinate, ethmoidal plate, upper third of nasal cavity (usually in olfactory cleft)	Can occur in all parts of the body

In summary, this reported case of DSRCT emphasizes the importance of incorporating DSRCT in the differential diagnosis of sinonasal tumors. And our study demonstrates the value of imunohistochemical analysis and molecular studies during the diagnosis of tumors which occur in an unusual location.

## Data Availability

All data generated or analyzed during this study are included in this published article.

## Abbreviations

CT: Computed tomography
DSRCT: Desmoplastic small round cell tumor
MRI: Magnetic resonance imaging
PENT: Primitive neurotodermal tumor
